# Relationship Between Standardized Test Scores and Board Certification Exams in a Combined Internal Medicine/Pediatrics Residency Program

**DOI:** 10.7759/cureus.13567

**Published:** 2021-02-26

**Authors:** Shelley R Ost, Daniel Wells, Patricia J Goedecke, Elizabeth A Tolley, Michael Kleinman, Natascha S Thompson

**Affiliations:** 1 General Internal Medicine, University of Tennessee Health Science Center College of Medicine, Memphis, USA; 2 General Pediatrics, University of Tennessee Health Science Center College of Medicine, Memphis, USA; 3 Preventive Medicine, University of Tennessee Health Science Center College of Medicine, Memphis, USA

**Keywords:** board certification, standardized test, in-training score, graduate medical education

## Abstract

Background

Combined Internal Medicine/Pediatrics (Med/Peds) residencies rely on categorical program data to predict pass rates for the American Board of Internal Medicine Certifying Exam (ABIM-CE) and the American Board of Pediatrics Certifying Exam (ABP-CE). There is insufficient literature describing what best predicts a Med/Peds resident passing board exams. In this study, we aimed to determine how standardized test scores predict performance on ABIM-CE and ABP-CE for Med/Peds residents.

Methodology

We analyzed prior exam scores for 91/96 (95%) residents in a Med/Peds program from 2008 to 2017. Scores from the United States Medical Licensing Examination (USMLE) Steps 1 and 2 Clinical Knowledge (CK) and In-Training Exams in Internal Medicine (ITE-IM) and Pediatrics (ITE-P) were analyzed with the corresponding ABIM-CE and ABP-CE first-time scores. Linear and logistic regression were applied to predict board scores/passage.

Results

USMLE 1 and 2 CK, ITE-IM, and ITE-P scores had a linear relationship with both ABIM-CE and ABP-CE scores. In the linear regression, adjusted R^2^ values showed low-to-moderate predictive ability (R^2^ = 0.11-0.35), with the highest predictor of ABIM-CE and ABP-CE being USMLE Step 1 (0.35) and Postgraduate Year 1 (PGY-1) ITE-IM (0.33), respectively. Logistic regression showed odds ratios of passing board certifications ranging from 1.05 to 1.53 per point increase on the prior exam score. The PGY-3 ITE-IM was the best predictor of passing both certifying exams.

Conclusions

In one Med/Peds program, USMLE Steps 1 and 2 and all ITE-IM and ITE-P scores predicted certifying exam scores and passage. This provides Med/Peds-specific data to allow individualized resident counseling and guide programmatic improvements targeted to board performance.

## Introduction

Combined Internal Medicine and Pediatrics residencies (Med/Peds) have maintained a prominent presence in graduate medical education for over 40 years [1]. After four years of training, graduates of Med/Peds residencies are eligible to sit for the certifying exams in both Internal Medicine (American Board of Internal Medicine Certifying Exam, ABIM-CE) and Pediatrics (American Board of Pediatrics Certifying Exam, ABP-CE). As of 2013, the ABP-CE examination is scored with a criterion-referenced set passing score independent of other takers [2]. The ABIM-CE is scored using a standard-setting procedure, with a set passing score determined by content experts [3]. Program accreditation relies on maintaining >5th percentile or at least 80% first-time pass rate on ABIM-CE and ABP-CE [4]. In categorical internal medicine and pediatrics literature, a significant amount of time and attention has been devoted to identifying the factors that are positively or negatively correlated with passing each of these exams.

Multiple studies in internal medicine have found positive correlation with board pass rate and In-Training Exams (ITE), while other studies have found correlation with United States Medical Licensing Exam (USMLE) Step 1 and number of overnight in-house calls in the final six months of training [5-8]. One study looked at program characteristics and determined that program features that contributed positively to pass rate were faculty-to-resident ratio, preliminary positions, a formal mentoring program, and on-site child care; the number of Doctors of Osteopathic Medicine (DO) in the program contributed negatively [9]. A recent study showed that research experience and awards such as Alpha Omega Alpha had no correlation to test scores or patient ratings; Step 2 Clinical Knowledge (CK) scores significantly correlated with patient ratings, ABIM-CE passage, and ITE, while Step 1 scores predicted ITE scores [10].

Pediatric literature has shown that ABP-CE passage was correlated positively with USMLE Step 1, USMLE Step 2 CK, combinations of Step 1 and 2, and ITE [11-13]. Program characteristics, such as faculty-to-resident ratio, average hours per week of lectures, and percent of US Allopathic Doctors of Medicine (MDs) are associated with higher pass rates for ABP-CE [9]. Chase et al. examined didactic and clinical factors related to ABP-CE passage and found that only the number of pediatric admissions correlated with improvement in ITE scores, without changing ABP-CE scores [14].

To our knowledge, no study has been conducted that looks solely at a Med/Peds program; therefore, Med/Peds programs have relied on categorical data to determine pass rate predictions and targets for early interventions with residents. In our study, we compiled all available standardized test data over a 10-year period from over 90 Med/Peds residents at the University of Tennessee Health Science Center (UTHSC) to determine which factors most accurately predicted performance on both ABIM-CE and ABP-CE in a Med/Peds residency.

## Materials and methods

We compiled test scores from graduates of the Med/Peds residency program at UTHSC from 2008 to 2017. Variables analyzed were USMLE Step 1, USMLE Step 2 CK, ITE data from all four years for both internal medicine and pediatrics, and certifying examination scores (pass/fail + numeric score when available). Board score data and ITE data are represented as percent correct, as is ITE data.

Univariate logistic and univariate linear regression methods were applied to data from 10 prior exams predicting performance on ABIM-CE and ABP-CE for residents graduating in internal medicine and pediatrics at UTHSC in years 2008 through 2017. To predict exam passage (pass/fail), logistic regression was applied. Linear regression was used to predict quantitative scores on board exams. The primary outcome in the logistic regression model was passing ABIM-CE and ABP-CE. The primary outcome in the linear regression model was correlation of scores between pre-board standardized testing and ABIM-CE and ABP-CE scores. While multivariable models using backward stepwise regression were developed in each case, due to high correlation among the predictive exams, each model reduced to one predictive variable. Demographics such as age, race, and gender were not included in the analysis as the goal was to develop standard prediction models that could be applied by any student.

Overall, 91 of 96 (95%) total subjects had pass/fail data for the ABIM-CE and 71 (74%) for the ABP-CE. Sixty-five subjects (68%) had quantitative scores for the ABIM-CE and 71 (74%) for the ABP-CE. Not all Med/Peds residents go on to take both certifying examinations, and some graduating in later years had not completed the examination at the time of the analysis. Residents with missing values only had the paired predictive/outcome variables for the missing results excluded from the analysis. Odds ratios with 95% confidence intervals and c-statistics were estimated for each prior exam with the certifying exams. Likewise, linear intercepts and coefficients, each with 95% confidence intervals, and adjusted R^2^ values were estimated for each prior exam with the two certifying exams.

In the linear regression models, we make the assumption that the relationship between each predictor and each predicted exam score is linear. In the logistic regression models, we assume that the logit is linear in the predictor exam scores. We have modeled the performance on the ABIM-CE and ABP-CE exams with the assumption that the meaningful step size is one point on any predictor exam. For example, for each increase of one point on a predictor exam scale, we expect the board exam score to increase by the amount reflected in the estimated linear regression coefficient. Or, we expect the odds of passing to increase by the odds ratio shown, per point change on the predictor exam.

The UTHSC Institutional Review Board approved the project with Exempt status.

## Results

Population

The mean USMLE Step 1 score for entering residents in our study was 216.3, with a median of 216. The mean Step 2 CK score was 227.3, with a median of 227. The percentage of US allopathic graduates ranged from a low of 64% in 2016 to a high of 100% in the classes of 2009, 2010, 2011, and 2015. Classes ranged from 27% to 80% female with an overall average of 51% female. Our ABIM-CE pass rate during the years of 2008-2017 was 89%, and our ABP-CE pass rate was 83%.

Results

The findings of logistic and linear regressions of prior exams with board exams are summarized in Tables 1, 2. Linear regression showed low-to-moderate predictive ability for each prior exam with relation to each certifying exam score. Logistic regression showed that an increase in score in each earlier exam increased the odds of passing each certifying exam.

**Table 1:** Predicting a passing score on ABIM-CE or ABP-CE board certifying exams using logistic regression of prior exams. ABIM: American Board of Internal Medicine; ABP: American Board of Pediatrics; USMLE: United States Medical Licensing Examination; CK: Clinical Knowledge; PGY: Postgraduate Years; ITE: In-Training Examination

Logistic regression predicting ABIM-CE^a^ pass							

Predictor	N	Odds ratio	Lower confidence interval	Upper confidence interval	c-statistic	P-Value
USMLE1 score	90	1.05	1.01	1.10	0.73	0.0241
USMLE2 score	81	1.06	1.02	1.11	0.82	0.0075
PGY1 Med	88	1.21	1.06	1.43	0.76	0.0127
PGY2 Med	87	1.22	1.09	1.43	0.78	0.0027
PGY3 Med	89	1.35	1.16	1.67	0.88	0.0011
PGY4 Med	90	1.28	1.11	1.56	0.84	0.0039
PGY1 Ped	89	1.14	1.01	1.31	0.67	0.0460
PGY2 Ped	89	1.11	1.01	1.24	0.68	0.0523
PGY3 Ped	87	1.12	1.02	1.24	0.71	0.0285
PGY4 Ped	87	1.11	1.01	1.25	0.70	0.0488
^a^ The American Board of Internal Medicine Certifying Exam					

Logistic regression predicting ABP-CE^b^ pass								
Predictor	N	Odds ratio	Lower confidence interval	Upper confidence interval	c-statistic	P-Value		
USMLE1 score	71	1.07	1.03	1.13	0.78	0.0041		
USMLE2 score	63	1.10	1.05	1.18	0.90	0.0008		
PGY1 Med	67	1.16	1.04	1.34	0.76	0.0210		
PGY2 Med	67	1.22	1.09	1.44	0.78	0.0031		
PGY3 Med	69	1.53	1.25	2.09	0.94	0.0009		
PGY4 Med	70	1.25	1.10	1.49	0.84	0.0029		
PGY1 Ped	70	1.29	1.11	1.57	0.80	0.0034		
PGY2 Ped	69	1.14	1.05	1.28	0.73	0.0074		
PGY3 Ped	67	1.28	1.12	1.52	0.85	0.0012		
PGY4 Ped	68	1.17	1.05	1.33	0.77	0.0069		
^b^ The American Board of Pediatrics Certifying Exam								

**Table 2:** Predicting ABIM-CE and ABP-CE certifying exam scores from prior exam scores using linear regression. ABIM-CE: American Board of Internal Medicine Certifying Exam; ABP: American Board of Pediatrics Certifying Exam; USMLE: United States Medical Licensing Examination; PGY: Postgraduate Years; CI: confidence interval; Hi: High; Coef: coefficient

Linear regression predicting ABIM-CE^a^ score										
Predictor	Intercept	Int CI Low	Int CI Hi	Coefficient	Coef CI Low	Coef CI Hi	R^2^	P-Value		
USMLE 1	-24.53	-53.56	4.50	0.39	0.26	0.52	0.35	< .001>		
USMLE 2	-20.98	-53.39	11.42	0.35	0.21	0.49	0.30	< .001>		
PGY1 Med	18.02	-2.16	38.20	0.78	0.40	1.15	0.22	< .001>		
PGY2 Med	3.80	-17.35	24.95	0.95	0.59	1.30	0.32	< .001>		
PGY3 Med	5.30	-16.35	26.96	0.85	0.52	1.19	0.30	< .001>		
PGY4 Med	-1.99	-23.93	19.94	0.91	0.59	1.23	0.34	< .001>		
PGY1 Ped	18.68	-4.21	41.57	0.76	0.34	1.18	0.18	< .001>		
PGY2 Ped	12.49	-11.69	36.68	0.75	0.37	1.13	0.20	< .001>		
PGY3 Ped	-2.61	-28.66	23.44	0.94	0.55	1.33	0.28	< .001>		
PGY4 Ped	-3.45	-29.99	23.09	0.93	0.55	1.32	0.28	< .001>		
^a^ The American Board of Internal Medicine Certifying Exam										

Linear regression predicting ABP-CE^b^ score										
Predictor	Intercept	Int CI Low	Int CI Hi	Coefficient	Coef CI Low	Coef CI Hi	R^2^	P-Value		
USMLE 1	29.61	10.10	49.12	0.19	0.10	0.28	0.20	< .001>		
USMLE 2	26.35	7.04	45.66	0.19	0.11	0.28	0.25	< .001>		
PGY1 Med	35.91	23.58	48.24	0.64	0.42	0.87	0.33	< .001>		
PGY2 Med	37.19	24.24	50.15	0.56	0.34	0.78	0.28	< .001>		
PGY3 Med	38.89	25.26	52.52	0.50	0.28	0.71	0.24	< .001>		
PGY4 Med	44.53	29.69	59.37	0.39	0.17	0.61	0.15	< .001>		
PGY1 Ped	47.79	32.25	63.33	0.40	0.12	0.68	0.11	0.0052		
PGY2 Ped	37.17	23.08	51.26	0.53	0.30	0.75	0.25	< .001>		
PGY3 Ped	30.07	12.81	47.34	0.61	0.35	0.87	0.25	< .001>		
PGY4 Ped	30.93	14.24	47.62	0.57	0.33	0.81	0.26	< .001>		
^b^ The American Board of Pediatrics Certifying Exam										

For the logistic regression, odds ratio estimates for the ABIM range from 1.05 to 1.35 on the prior exams, and for the ABP from 1.07 to 1.53. Using the c-statistic for comparison, the PGY-3 ITE in internal medicine is the best predictor of passing either the ABIM-CE exam or the ABP-CE at 0.86 and 0.94, respectively (see Table 1).

**Figure 1:**
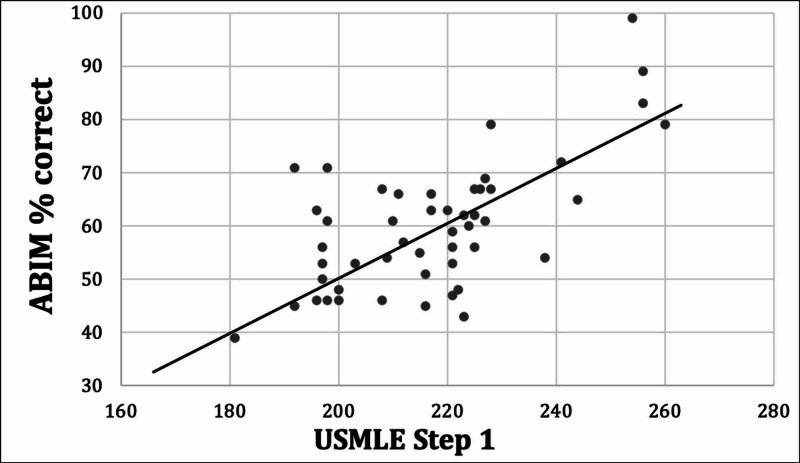
USMLE Step 1 versus ABIM linear regression. USMLE: United States Medical Licensing Exam; ABIM: American Board of Internal Medicine

**Figure 2:**
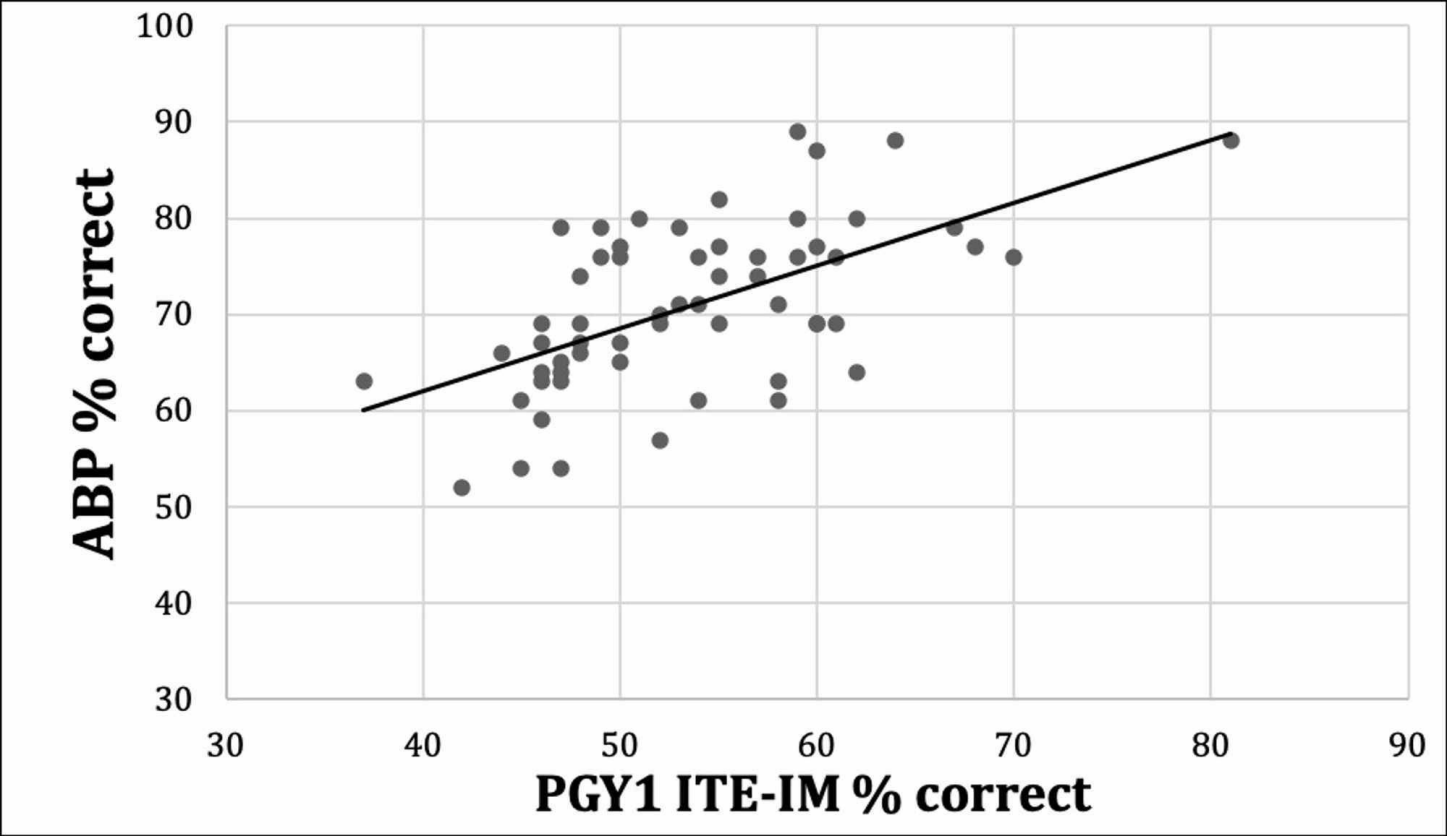
PGY-1 ITE-IM versus ABP-CE linear regression. PGY: Postgraduate Year; ITE: In-Training Examination; IM: Internal Medicine; ABP: American Board of Pediatrics; CE: Certifying Examination

Among the linear regressions, squared Pearson correlation coefficient (R^2^) values for the ABIM-CE range from 0.18 to 0.35, and for the ABP from 0.11 to 0.33 (see Table 2), showing low-to-moderate predictive ability. According to these R^2^ values, the USMLE Step 1 exam is the most effective predictor of quantitative score on the ABIM-CE, while the PGY-1 exam in internal medicine is the most effective predictor of the ABP-CE score. See Figure 1 for the linear regression plot for the USMLE Step 1 versus ABIM-CE percent correct, and Figure 2 for the linear regression plot of the PGY1 IM ITE versus ABP-CE percent correct.

## Discussion

Our results show that USMLE Step 1, Step 2 CK, and all years of both internal medicine and pediatrics ITE scores predict with varying degrees of reliability the numeric ABP-CE and ABIM-CE scores and odds ratios for passing the certifying examinations. Though presumably the knowledge base for internal medicine and pediatrics is different, there was still correlation between internal medicine ITE scores and ABP-CE score, as well as between pediatrics ITE and ABIM-CE scores. Correlation of scores across specialties implies that generalizable factors such as knowledge base, study skills, or test-taking skills significantly affect results. Ours is the first study to compare the test scores on exams from the two specialties, and the first Med/Peds program-specific study of test scores.

Linear regression analysis showed low-to-moderate predictive ability for all tests analyzed with respect to predicting numeric certifying exam scores. Previous internal medicine studies including Brateanu et al. also found a linear correlation between ITE scores and ABIM scores [7], though their data showed a higher correlation between PGY-2 and PGY-3 ITEs with ABIM-CE. Kay et al. found a similar moderate correlation between USMLE Step 1 and ABIM-CE, as well as between ITE-IM exams and ABIM-CE [8]. McDonald et al. and Perez and Greer also showed correlation between USMLE and ITE scores in internal medicine [15,16], but did not extend their study to certifying examination scores. Work in other specialties related USMLE scores to in-training scores in similar medical specialties such as emergency medicine and dermatology [17,18]. In-training scores in obstetrics and gynecology, psychiatry, emergency medicine, and family medicine have been shown to correlate with passage of the corresponding certifying examinations [19-23].

The unexpected finding that earlier tests such as USMLE Step 1 (taken during medical school) and the PGY-1 internal medicine ITE (taken in September of PGY-1 year) correlate more highly with certifying exam scores than later ITE scores implies that other factors not accounted for by our study have a significant impact, such as fatigue or test-taking skills.

Limitations

Limitations include that our study was performed in only one residency program, which allowed local systems factors to impact our trainees’ test scores. In addition, some scores were unavailable, limiting the data set and reducing the statistical power. Our statistical analysis attempted to correct for year taken, but the significant change in scoring of the ABP-CE still may have affected the results. The change in USMLE Step 1 to pass/fail in 2022 will limit future use of scores from the USMLE Step 1 in predicting certification exam passage. Finally, the percent correct was used for the ITE and certifying exams, and the percent correct score that is a passing score on the certifying examinations varies.

Future directions

Our data can be used to target board study or test-taking skills interventions to individuals at higher risk of board failure within a Med/Peds program. Weekly board study emails and mandatory reading and board study group sessions have been shown to have significant correlation with passage of the ABP certifying exam, with a greater effect among residents with lower pediatrics ITE scores [24,25]. In another study, increased pediatric admissions was found to improve ITE scores [14], suggesting that optimal clinical exposure contributes to improving medical knowledge scores.

In the future, we plan to strengthen our score predictions with upcoming graduates’ data. We hope to involve other programs to broaden our source of data and improve the accuracy of our predictions. The program will use the prediction tool to counsel residents at risk, and to recommend a more intensive board review strategy similar to the studies mentioned above for those residents to improve their odds of passing their board exams.

## Conclusions

Our study confirms that in combined Med/Peds programs, USMLE scores and ITE scores show low-to-moderate correlation with future board certification exam scores. Furthermore, there is low-to-moderate correlation even between internal medicine ITE scores and pediatrics certifying examination scores, and vice versa. USMLE and ITE scores can help predict future certification exam passage and scores, with varying degrees of accuracy. Finally, the R^2^ values for prior tests compared to ABIM-CE were generally higher than those compared with ABP, indicating more variability in ABP-CE scores compared to ABIM-CE scores.
